# A Unique Subset of γδ T Cells Expands and Produces IL-10 in Patients with Naturally Acquired Immunity against Falciparum Malaria

**DOI:** 10.3389/fmicb.2017.01288

**Published:** 2017-07-19

**Authors:** Tomoyo Taniguchi, Kaiissar Md Mannoor, Daisuke Nonaka, Hiromu Toma, Changchun Li, Miwako Narita, Viengxay Vanisaveth, Shigeyuki Kano, Masuhiro Takahashi, Hisami Watanabe

**Affiliations:** ^1^Department of Parasitology, Graduate School of Medicine, Gunma University Maebashi, Japan; ^2^Center for Medical Education, Graduate School of Medicine, Gunma University Maebashi, Japan; ^3^Immunobiology Group, Center of Molecular Biosciences, Tropical Biosphere Research Center, University of the Ryukyus Nishihara, Japan; ^4^Department of Pathology, University of Maryland School of Medicine, Baltimore MD, United States; ^5^Department of Parasitology and Immunopathoetiology, Graduate School of Medicine, University of the Ryukyus Nishihara, Japan; ^6^Department of Health Sciences, Trans-disciplinary Research Organization for Subtropics and Island Studies, University of the Ryukyus Nishihara, Japan; ^7^Laboratory of Hematology and Oncology, Graduate School of Health Sciences, Niigata University Niigata, Japan; ^8^Center for Malariology, Parasitology and Entomology Vientiane, Laos; ^9^Research Institute, National Center for Global Health and Medicine Tokyo, Japan; ^10^Infectious Diseases Research Center of Niigata University in Myanmar, Institute of Medicine and Dentistry, Niigata University Niigata, Japan

**Keywords:** γδ T cells, naturally acquired immunity, *Plasmodium falciparum*, falciparum malaria, IL-10

## Abstract

Although expansions in γδ T cell populations are known to occur in the peripheral blood of patients infected with *Plasmodium falciparum*, the role of these cells in people with naturally acquired immunity against *P. falciparum* who live in malaria-endemic areas is poorly understood. We used a cross-sectional survey to investigate the role of peripheral blood γδ T cells in people living in Lao People’s Democratic Republic, a malaria-endemic area. We found that the proportion of non-Vγ9 γδ T cells was higher in non-hospitalized uncomplicated falciparum malaria patients (UMPs) from this region. Notably, we found that the non-Vγ9 γδ T cells in the peripheral blood of UMPs and negative controls from this region had the potential to expand and produce IL-10 and interferon-γ when cultured in the presence of IL-2 and/or crude *P. falciparum* antigens for 10 days. Furthermore, these cells were associated with plasma interleukin 10 (IL-10), which was elevated in UMPs. This is the first report demonstrating that, in UMPs living in a malaria-endemic area, a γδ T cell subset, the non-Vγ9 γδT cells, expands and produces IL-10. These results contribute to understanding of the mechanisms of naturally acquired immunity against *P. falciparum*.

## Introduction

Malaria is caused by protozoan parasites of the genus *Plasmodium* and is widespread in tropical and subtropical regions of the world. Approximately half the world’s population is at risk of malaria, and 148–304 million cases of malaria and 0.2–0.6 million associated deaths are estimated to occur each year (World Health Organization, 2016). There is still no effective vaccine for malaria (Langhorne et al., 2008; Riley and Stewart, 2013), thus posing a problem for those exposed to *Plasmodium falciparum*, the species that causes the most severe form of the disease. An effective malaria vaccine must induce long-lasting protective immunity. However, it is difficult to induce sufficient immunological memory against human malarial parasites, because of their high levels of antigenic polymorphism and complex parasitic life cycle (Covell et al., 1953; Healer et al., 2004). Additionally, the life span of plasma cells in humans is short (Dorfman et al., 2005; Akpogheneta et al., 2008; Portugal et al., 2013). Nevertheless, naturally acquired immunity, which occurs after repeated infections with a parasite, does develop in people living in malaria-endemic areas; although this immunity is incomplete, it decreases clinical disease and lowers the density of parasitemia (Doolan et al., 2009). For effective vaccine development, it is important to study individuals who have developed a naturally acquired immunity against malaria.

γδ T cells are T cells carrying the γδ T cell receptor (TCR), and their system of antigen recognition differs from that of αβ T cells. These cells have drawn much attention in relation to their involvement in host defense against infection and tumors, although they constitute a very small population of cells in the peripheral blood. γδ T cells perform a variety of functions, including cytotoxic functions and initiation of acquired immunity (Bonneville et al., 2010; Vantourout and Hayday, 2013). Before γδ T cells were discovered, human peripheral blood mononuclear cells (PBMCs) had been reported to proliferate in the culture supernatants and extracts of malaria-infected erythrocytes *in vitro* (Greenwood and Vick, 1975). After the concept of the γδ T cell population had been established, it was confirmed that splenic γδ T cell populations increase during malaria infection in both humans and mice (Minoprio et al., 1989; Bordessoule et al., 1990). There have been many conflicting reports on whether γδ T cells and their subsets increase after malaria infection. Some reports claim that in patients with primary or acute falciparum malaria, γδ T cells increase after antimalarial treatment and that this increase persists for 3–4 weeks after treatment (Ho et al., 1990; Roussilhon et al., 1990; Chang et al., 1992; Hviid et al., 1996, 2001; Schwartz et al., 1996; Worku et al., 1997). However, there are some reports showing that no increase occurs in γδ T cells in the peripheral blood of UMPs from endemic areas (Goodier et al., 1993; Hviid et al., 1996). We have previously shown that unconventional T cells, including γδ T cells, are associated with protection against malaria in murine models of the disease (Weerasinghe et al., 2001; Mannoor et al., 2002; Bakir et al., 2006; Taniguchi et al., 2007; Li et al., 2012). We have also observed both the presence and absence of an increase in γδ T cells in peripheral blood samples from malaria patients in Southeast Asia (Watanabe et al., 2003). Recently, there have been reports that repeated malaria infection in malaria-endemic area is associated with a decreased percentage of Vδ2 γδ T cells in the peripheral blood and decreased proliferation and cytokine production in response to malarial antigens (Jagannathan et al., 2014; Farrington et al., 2016).

We, therefore, hypothesized that γδ T cells, which increase in primary or acute infections, do not increase in people with naturally acquired immunity to malaria. To evaluate this hypothesis and to investigate the role of γδ T cells in people with naturally acquired immunity against *P. falciparum* in more detail, we analyzed the dynamics of γδ T cells in patients with falciparum malaria living in the Lao People’s Democratic Republic, where malaria is endemic. We found that a γδ T cell subset, the non-Vγ9 γδT cells, which increases in malaria patients living in endemic areas, may play an important role in the acquisition of natural immunity.

## Materials and Methods

### Ethics Statement

This study was approved by the National Ethics Committee for Health Research, Ministry of Health, Lao People’s Democratic Republic (PDR) and the Ethics Review Board of the University of the Ryukyus, Japan. Informed consent was obtained from each participant in the study. All procedures followed were in accordance with the ethical standards of the responsible committee on human experimentation (institutional and national) and with the Helsinki Declaration of 1964 and later revision. Identifying information of patients of human subjects, including names, initials, addresses, or any other data that might identify patients do not be included in written descriptions in this article. Informed consent from minors was obtained from their parent before sample collection.

### Study Site and Population

This cross-sectional survey was conducted at the end of each rainy season from 2005 to 2008 in villages of the Phouvong District of Attapeu Province, an area with high malaria endemicity in Lao PDR^1^. The annual incidence of malaria in 2008 in this province was 14.3 cases per 1,000 people, which is the second highest incidence of malaria in the country (Jorgensen et al., 2010). Non-endemic controls (NECs) were recruited voluntarily from the population in Vientiane, the capital of Lao PDR, and Japanese healthy controls (JHCs) were recruited from volunteers in Okinawa Prefecture, Japan.

### Blood Samples

Falciparum malaria was diagnosed at the primary schools in the villages or the village head’s house. All subjects were assessed with a rapid immunochromatographic test (ICT; Paracheck Pf^®^, Orchid Biomedical Laboratories, Goa, India) and a blood smear analysis. After the diagnostic tests were performed, heparinized blood samples (4 mL) were collected from the *P. falciparum*-positive subjects and some *P. falciparum*-negative volunteers (NCs) as controls. All *P. falciparum*-positive subjects with uncomplicated falciparum malaria, lacking signs of organ compromise or other severe symptoms, were classified as non-hospitalized uncomplicated malaria patients (UMPs), according to the World Health Organization guidelines (World Health Organization, 2006). Parasitemia and species identity were assessed by microscopic observation of the blood smears. The data from *P. vivax*-infected subjects and false-positive (ICT positive but microscopic observation-negative) subjects were excluded from the analysis. *P. falciparum*-positive subjects were treated with Coartem^®^ (Novartis Pharma, Basel, Switzerland), an artemisinin-based combination therapy. After the number of white blood cells was counted, the plasma was separated from the blood by centrifugation. PBMCs were obtained by Ficoll–Paque (GE Healthcare Life Sciences, Uppsala, Sweden) gradient centrifugation. The plasma and PBMCs from *P. falciparum*-positive and -negative subjects were initially stored in liquid nitrogen (-196°C) and at -80°C until analysis.

### Antibody Assays

Enzyme-linked immunosorbent assays (ELISAs) of anti-*P. falciparum* IgG antibodies (Abs) were performed as below. Each well of a 96-well plate was coated with 0.5 μg of crude *P. falciparum* FCR-3 antigen extract (Pf Ag) in 100 μL of 50 mM Na_2_CO_3_ buffer, a kind gift from Dr. S. Nakazawa (Department of Protozoology, Institute of Tropical Medicine, NEKKEN, and the Global COE Program, Nagasaki University, Nagasaki, Japan). The plates were incubated overnight at 4°C to allow antigen binding, after which the wells were washed four times with phosphate-buffered saline (PBS) containing 0.05% Tween 20 (PBS/T) and then blocked for 2 h at 37°C with 150 μL of PBS/T containing 1% bovine serum albumin (BSA; Nacalai Tesque, Kyoto, Japan) per well. Test sera (100 μL aliquots), each diluted 1:400 in PBS/T containing 1% BSA, were added in duplicate, and the plates were incubated for 1 h at room temperature. The wells were washed four times with PBS/T and then incubated for 30 min at room temperature with 100 μL of horseradish-peroxidase-conjugated goat anti-human IgG (Promega, Madison, WI, United States) diluted 1:2500 in PBS/T. The wells were washed four times with PBS/T, 100 μL of tetramethylbenzidine substrate (TMB One Solution; Promega) was added, and the plates were incubated at room temperature for up to 15 min before the reaction was stopped with 100 μL of 0.5 M H_2_SO_4_. The optical density (OD) of the wells at 450 nm (OD_450_) was determined with a microplate reader. To generate standardized units of specific IgG reactivity, a standard curve was generated to allow the OD units to be interpolated to linearized units of antibody concentration. If a sample OD_450_ exceeded 2.0, the sample was diluted further as soon as possible to achieve an OD_450_ within the range of 0.0–2.0.

### Flow Cytometric Analysis

Frozen PBMCs were thawed quickly for analysis. The surface phenotypes of the lymphocytes were determined, and the intracellular cytokine measurements were made with three- or four-color immunofluorescence tests. Fluorescein isothiocyanate (FITC), phycoerythrin (PE), or biotin-conjugated monoclonal antibodies (mAbs) were used, and biotin-conjugated reagents were developed using allophycocyanin-conjugated streptavidin (Caltag Laboratories, Burlingame, CA, United States). Anti-CD3 (145-2C11), anti-Vγ9 TCR (TM-β1), anti-αβ TCR (H57-597), anti-γδ TCR (GL3), anti-IL-10, and anti-interferon γ (IFN-γ) mAbs (BD Biosciences, Mountain View, CA, United States) and anti-Vδ1 TCR (R9.12) mAb (Beckman Coulter, Marseille, France) were used. The cells were examined with a FACSCalibur flow cytometer (BD Biosciences). Dead cells were excluded with forward scatter, side scatter, and propidium iodide gating.

### RT-PCR Analysis

Total RNA was extracted from the PBMCs of UMPs and NECs with the RNeasy Plus Mini Kit (Qiagen GmbH, Hilden, Germany). To detect the TCRγ and δ chain mRNAs, the cDNA was synthesized from 2.5 μg of RNA with random primers and Superscript VILO reverse transcriptase (Invitrogen, Carlsbad, CA, United States). The cDNA was amplified with sense primers specific for TCR Vγ or Vδ and antisense primers specific for TCR Cγ or Cδ, as previously described (Kageyama et al., 1994; Boria et al., 2008). The PCR products were visualized under UV illumination on 2% agarose gels stained with ethidium bromide.

### PBMC Culture and Intracellular Cytokine Staining

Thawed PBMCs (1–2 × 10^6^ cells/mL) were cultured with or without interleukin 2 (IL-2, 50 U/mL) or IL-2 and Pf Ag (5 μg/mL) for 10 days at 37°C under 5% CO_2_. The cultured PBMCs were restimulated with Leukocyte Activation Cocktail (BD Biosciences) containing the phorbol ester phorbol 12-myristate 13-acetate, a calcium ionophore (ionomycin), and brefeldin A for 4 h at 37°C under 5% CO_2_. The cells were then fixed, permeabilized, stained with 20 μL of PE-conjugated rat anti-human IL-10 antibody or mouse anti-human IFN-γ antibody (BD Biosciences), and analyzed with a FACSCalibur flow cytometer (BD Biosciences).

### Cytokine Detection

The cytokines in the plasma and culture supernatants were measured using a BD^TM^ Cytometric Bead Array Flex Set and BD^TM^ Human Soluble Protein Master Buffer kit according to the manufacturer’s instructions (BD Biosciences). Aliquots (50 μL) of the test sera, diluted 1:5 with dilution buffer, were used. IL-1β, IL-2, IL-4, IL-6, IL-8, IL-10, IL-12p70, tumor necrosis factor α, and IFN-γ were acquired on a FACSCalibur flow cytometer (BD Biosciences) and analyzed using FCAP Array software (BD Biosciences).

### Statistical Analysis

The study subjects were matched for age and sex. The Mann–Whitney *U*-test was used for two-group comparisons, the Kruskal–Wallis test was used for three-group comparisons, and paired and unpaired *t*-tests were used to analyze whole PBMC cultures. Spearman’s rank correlation was used for correlational analyses. All analyses were performed with Prism version 6.0 (GraphPad Software, La Jolla, CA, United States) and JMP version 8 statistical software (SAS, Cary, NC, United States).

## Results

### Expansion of a Unique γδ T Cell Subset, the Non-Vγ9 γδ T Cells, in Falciparum Malaria Patients

Despite many reports of γδ T cells in malaria patients, it is unclear whether γδ T cells are induced during malarial infection (Ho et al., 1990; Roussilhon et al., 1990; Goerlich et al., 1991; Currier et al., 1992; Goodier et al., 1992; Zevering et al., 1992; Dick et al., 1996; Hviid et al., 1996, 2001; Rzepczyk et al., 1996; Goodier and Targett, 1997; Worku et al., 1997; Waterfall et al., 1998). To investigate this phenomenon in more detail, PBMCs and plasma obtained from UMPs and NCs living in malaria-endemic areas of Attapeu Province, Lao PDR and from NECs in Vientiane, the country’s capital, were analyzed during physical examination of the subjects. The hematological characteristics of the UMPs, NCs, and NECs in this study are summarized in **Table 1** and Supplementary Figure S1. In agreement with the high incidence of malaria (Jorgensen et al., 2010), all subjects living in the Phouvong District of Attapeu Province had anti-*P. falciparum* IgG Abs in their plasma, and the subjects in the NC group had a previous history of natural malaria infection. The anti-*P. falciparum* IgG Ab titers were higher in the UMPs (median 827.9, range 86.9–8685.6) than those in the NCs (median 462.1, range 7.0–10,097.3; *p* < 0.0001). The UMPs living in the malaria-endemic area included both symptomatic (body temperature ≥ 37.5°C) and asymptomatic falciparum malaria patients (parasitemia < 250,000/μL, hematocrit > 15%) (World Health Organization, 2006).

**Table 1:** Hematological characteristics of patients with falciparum malaria and negative controls.

Characteristic	UMPs^a^ (*n* = 137)	NCs (*n* = 31)	NECs (*n* = 65)	*p-*Value^b^
Age, median (range)	10.0 (0.5–45)	15.0 (5–31)	12.0 (5–20)	
Sex (female/male)	66/71	20/11	33/32	
WBC/μL, median (range)	8,500 (3,500–16,400)	10,400 (5,800–16,400)	7,500 (3,500–13,500)	<0.0001
% Neutrophil, median (range)	42.3 (14.0–88.0)	44.0 (13.5–72.5)	46.0 (24.5–72.5)	0.9103
% Lymphocyte, median (range)	38.5 (7.0–75.0)	37.5 (21.5–85.5)	47.0 (23.5–70.0)	0.0037
% Monocyte, median (range)	3.0 (0.0–16.0)	3.0 (0.0–8.5)	1.5 (0.0–10.0)	0.001
% Eosinophil, median (range)	9.3 (0.0–37.5)	11.5 (0.0–28.0)	5.0 (0.0–23.5)	0.0002
% Basophil, median (range)	0.0 (0.0–1.5)	0.0 (0.0–1.0)	0.0 (0.0–1.0)	0.0592
Parasitemia/μL, median (range)	640 (32–180,000)	(-)	(-)	
Microscopic observation *(Pf)*	(+)	(-)	(-)	
*α-Pf* IgG Abs titer, median (range)	827.9 (86.9–8685.6)	462.1 (7.0–10097.3)	0.0 (0.0–1307.1)	<0.0001

*UMPs, uncomplicated malaria patients; NCs, negative controls; NECs, non-endemic controls; Pf, *Plasmodium falciparum*; Abs, antibodies. ^a^Diagnosed using Pf rapid test and microscopic observation and excluded Mix (Pf and P. vivax) infection and Rapid test (+)/Microscopic observation (-). ^b^Calculated from Kruskal–Wallis test.*

**FIGURE 1:**
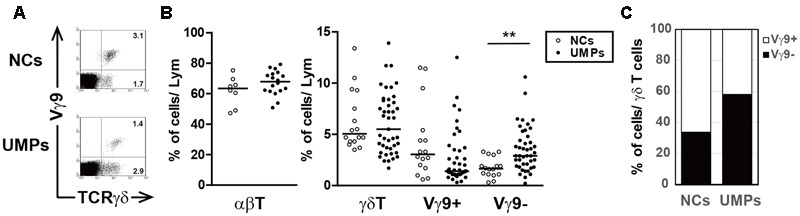
Phenotypic characterization of γδ T cells in falciparum malaria patients. **(A)** Flow cytometric profiles of γδ T cell subsets in negative controls (NCs) and non-hospitalized uncomplicated malaria patients (UMPs) from Lao PDR. The numbers in the figure represent the median percentages of immunofluorescence-positive cells. Two-color staining was performed with mAbs directed against γδ TCR and Vγ9 TCR. **(B)** The proportion of αβ T cells and γδ T cell subsets in 8–16 NCs (○) and 18–43 UMPs (●). **(C)** The proportion of γδ T cell subsets in γδ T cells 8–16 NCs (○) and 18–43 UMPs (●). Statistical analysis was performed with the non-parametric Mann–Whitney *U*-test **(B)**. ^∗∗^*p* < 0.01.

The lymphocytes in the PBMCs were phenotypically characterized by using flow cytometry. As in other acute infections, acute falciparum malaria causes a decrease in lymphocyte counts (Supplementary Table S1 and Figure S2). A decrease in lymphocyte numbers in UMPs inhabiting the malaria-endemic area and a significant increase in white blood cell numbers in NCs were observed (**Table 1** and Supplementary Figure S3); therefore, the percentage of cell populations was used for comparisons. The change in lymphocyte cell subsets was minimal, and the percentage of γδ T cells (UMPs: median 5.5%, range 1.2–13.9%; NCs: median 5.1%, range 3.5–13.4%, *p* = 0.6208) and αβ T cells (UMPs: 67.9%, 50.8–79.2%; NCs: 63.4%, 47.2–75.3%, *p* = 0.1917) was not higher in the UMPs than in the NCs (**Figure 1** and Supplementary Figure S4). However, a specific γδ T cell subset, the non-Vγ9 γδ T cells (UMPs: 2.9%, 0.2–10.6%; NCs: 1.7%, 0.3–3.3%, *p* = 0.0018), was expanded in the UMPs (**Figure 1B**), and the proportion of non-Vγ9 γδ T cells within the γδ T cell subset (UMPs: 58.1%, 10.7–92.7%; NCs: 33.7%, 8.8–82.5%, *p* = 0.0025) was also higher in the UMPs (**Figure 1C**). These results differed from those of hospitalized patients with severe falciparum malaria, in whom the Vγ9 γδ T cell subset increased (Supplementary Table S1 and Figures S5A,B). Although there was no change in B cell subsets between UMPs and NCs, we found a significant increase in B cells and memory B cells (IgG+CD27-CD20+) in UMPs compared with those in NECs (Supplementary Figure S6).

### γδ TCR Usage by γδ T Cells in Patients with Falciparum Malaria

**FIGURE 2:**
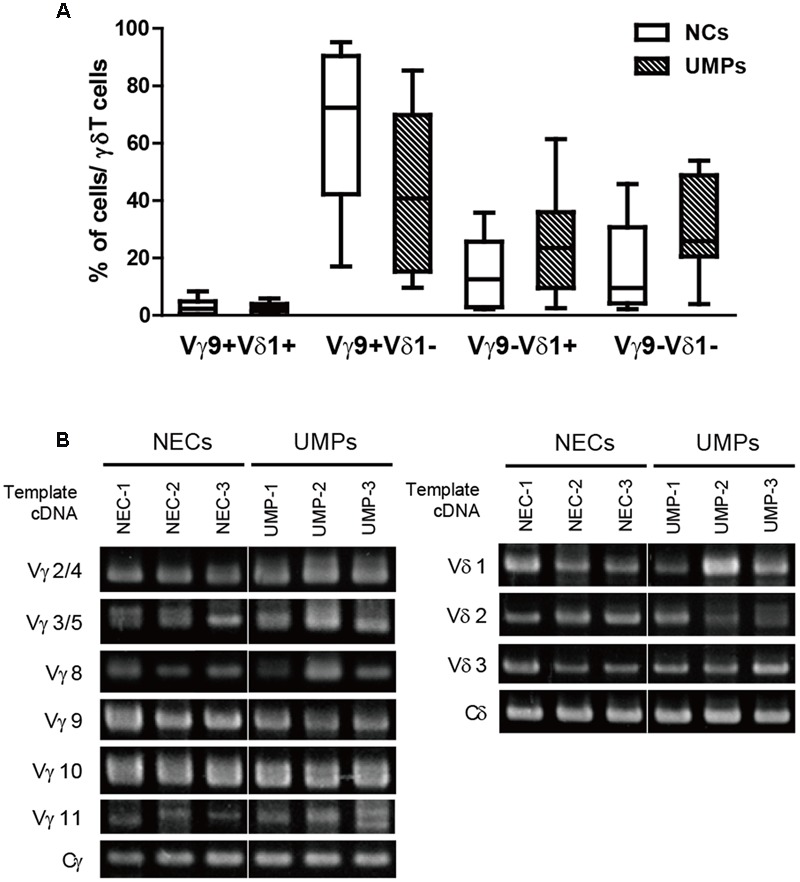
Identification of the TCR variable region repertoire in γδ T cells in falciparum malaria patients. **(A)** The percentages of Vγ9^+/-^ or Vδ1^+/-^ cells in γδ T cells from negative controls (NCs, □) and non-hospitalized uncomplicated malaria patients (UMPs, ■) determined with flow cytometry. Three-color staining was performed with mAbs against γδ TCR, Vγ9 TCR, and Vδ1 TCR. **(B)** Identification of the TCR variable region repertoire of γδ T cells in falciparum malaria patients via RT-PCR. The levels of Vγ2/4, Vγ3/5, Vγ8, Vγ9, Vγ10, Vγ11, and Vδ1–3 were determined in non-endemic controls (NECs) and UMPs. Representative results of three experiments are shown.

γδ T cells with non-TCR Vγ9 chains were increased in the UMPs, and the other major subset in the peripheral blood had the Vδ1 chain; however, the predominant γδ T cell subset was Vγ9Vδ2. Therefore, using flow cytometry, we examined whether these non-Vγ9 γδ T cells had Vδ1 chains. The γδ T cell subsets that were elevated in the UMPs living with endemic malaria were Vγ9^-^Vδ1^+^ (UMPs: 21.4%, 2.5–61.4%; NCs: 12.6%, 2.2–35.8%) and Vγ9^-^Vδ1^-^ (UMPs: 25.3%, 3.9–53.9%; NCs: 9.5%, 2.2–45.8%). In contrast, Vγ9^+^Vδ1^-^ cells were the predominant subset in the NCs, but there was no significant difference in γδ TCR usage by γδ T cells between NCs and UMPs, as determined by flow cytometry (**Figure 2A**). Therefore, we performed further analysis of γδ TCR usage by γδ T cells from UMPs compared with that in NECs by using RT-PCR (**Figure 2B**). We found that Vγ and Vδ were detected at the same level in the UMPs and NECs, and the Vγ9 levels tended to be lower in the UMPs than in the NECs, whereas the levels of Vγ2/Vγ4, Vγ3/Vγ5, Vγ8, Vγ11, Vδ1 and Vδ3 tended to be higher in the UMPs. Thus, γδ TCR repertoire in the non-Vγ9 γδ T cells was highly polyclonal in the UMPs living in the malaria-endemic area, although the Vγ9Vδ2 T cell subset was predominant in the NCs and NECs.

### IL-10 and IFN-γ Production by the Expanding Non-Vγ9 γδ T Cells

**FIGURE 3:**
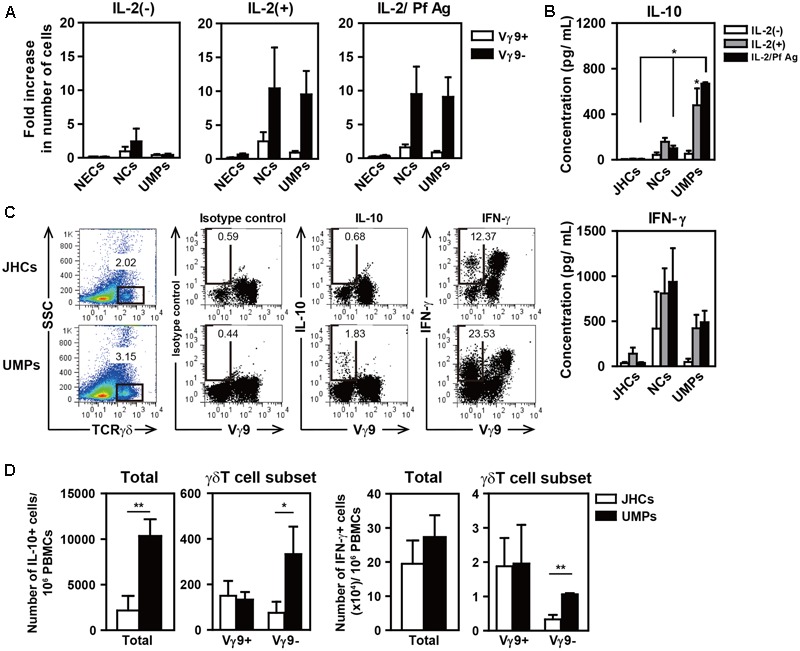
High levels of IL-10 production in the culture supernatants of PBMCs from patients with uncomplicated malaria in the presence of IL-2 and Pf Ag for 10 days. Fold increase in the number of γδ T cell subsets **(A)** and the production of IL-10 and IFN-γ **(B)** after the culture of PBMCs from NECs, Japanese healthy controls (JHCs), negative controls (NCs), and non-hospitalized uncomplicated malaria patients (UMPs). PBMCs (1–2 × 10^6^ cells/mL) were cultured in medium containing 10% fetal calf serum with or without IL-2 (50 U/mL) or IL-2/Pf Ag (5 μg/mL) for 10 days. Cytokines in the culture supernatants were detected with a CBA Flex Set assay. Flow cytometric profiles of intracellular IL-10 and IFN-γ staining in γδ T cells **(C)**, and the numbers of IL-10-producing (**D**, left) and IFN-γ-producing cells (**D**, right) in the total PBMCs and γδ T cell subsets per 10^6^ PBMCs from 3–5 JHCs and 3–5 UMPs after culture for 10 days. Three-color staining was performed with mAbs directed against γδTCR, Vγ9TCR, IL-10, and IFN-γ. The numbers in the figures represent the mean percentages of immunofluorescence-positive cells. Representative results of three experiments are shown. Statistical analyses were performed with one-way ANOVA with Dunnett’s *post hoc* test **(B)** and unpaired *t*-test **(D)**. ^∗^*p* < 0.05, ^∗∗^*p* < 0.01.

The role of γδ T cells in malaria infection remains unclear. However, it has been reported that these cells contribute to host defense and disease pathology during infections (Stevenson and Riley, 2004; Schofield and Grau, 2005). To investigate the function of non-Vγ9 γδ T cells after *P. falciparum* infection, we cultured whole PBMCs from falciparum malaria patients in the presence of IL-2 and crude Pf Ag for 10 days. The cytokine concentrations were measured in the culture supernatants because it is difficult to culture non-Vγ9 γδ T cells; these cells constitute a very small population of cells in the peripheral blood, and the specific antigen recognized by the TCR remains unknown. Surprisingly, non-Vγ9 γδ T cells from NCs and UMPs in the malaria-endemic area increased 10-fold in the presence of IL-2 or IL-2 and Pf Ag after 10 days of culture (**Figure 3A**). Moreover, high levels of IL-10 were detected in the culture supernatants of PBMCs from the malaria-endemic groups (**Figure 3B**). The highest IL-10 production was observed in the supernatants of PBMCs from UMPs in the presence of IL-2 and/or Pf Ag. Interestingly, high concentrations of IFN-γ were also detected in those supernatants.

To determine whether non-Vγ9 γδ T cells produce IL-10 in patients with falciparum malaria, we used intracellular staining for IL-10 and confirmed that IL-10 was indeed produced in non-Vγ9 γδ T cells after whole PBMCs were cultured for 10 days. We found that non-Vγ9 γδ T cells still produced IL-10, and higher levels of IL-10-producing cells were present in the PBMCs from UMPs than in those from JHCs, although many other IL-10-producing cells were present (**Figures 3C,D**). It has been reported that IFN-γ- or IL-17-producing Vγ9 γδT cells can be identified by using the cell surface marker CD27 (Ribot et al., 2009, 2010; Ribot and Silva-Santos, 2013). Approximately 80% of Vγ9 γδ T cells in the present study were CD27-positive, but the classification of non-Vγ9 γδ T cells by CD27 expression was difficult (data not shown).

We found that the non-Vγ9 γδ T cells from patients living with recurrent malarial infections responded to IL-2 and/or crude Pf Ag, and these cells proliferated and produced both of IFN-γ and IL-10. Both of these are important cytokines for protecting the host against malarial infection. IFN-γ production by non-Vγ9 γδ T cells was also found in the JHCs with no history of malaria, although no proliferation of non-Vγ9 γδT cells or IL-10 production was observed.

### Proportion of Non-Vγ9 γδ T Cells in Falciparum Malaria Patients Correlates with Plasma Levels of IL-10

**FIGURE 4:**
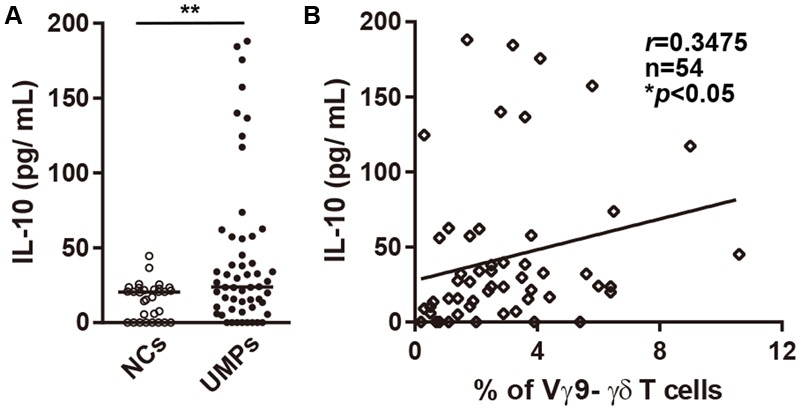
Plasma levels of IL-10 in falciparum malaria patients. **(A)** Plasma levels of IL-10 in negative controls (NCs, ○) and non-hospitalized patients with uncomplicated malaria (UMPs, ●). **(B)** Correlation between plasma levels of IL-10 and the percentage of non-Vγ9 γδ T cells in UMPs. *r, n*, and *p* values are shown (upper right). Statistical analyses were performed with the Mann–Whitney *U*-test **(A)** and Spearman’s rank correlation **(B)**. ^∗^*p* < 0.05, ^∗∗^*p* < 0.01.

To confirm whether non-Vγ9 γδ T cells correlate with levels of cytokines including IL-10 and IFN-γ in peripheral blood, we examined the plasma levels of cytokines in the infected patients living in endemic areas. The plasma levels of IL-10 were significantly higher in UMPs than in NCs (UMPs: median 28.3 pg/mL, range 11.0–664.8 pg/mL; NCs: 21.9 pg/mL, 14.5–44.5 pg/mL, *p* = 0.0079; **Figure 4A**). None of the other cytokines (IL-1β, IL-2, IL-4, IL-6, IL-8, IL-12p70, tumor necrosis factor α, and IFN-γ) were elevated in UMPs living in endemic areas (Supplementary Table S2). These results were consistent with the mild symptoms of falciparum malaria in patients living in endemic areas. When we examined the relationship between plasma levels of cytokines and non-Vγ9 γδ T cells proportion, we found that the plasma levels of IL-10 correlated with non-Vγ9 γδ T cell proportion (*r* = 0.3475, *n* = 54, *p* = 0.0100; **Figure 4B**) but that of IFN-γ did not. These data suggested that non-Vγ9 γδ T cells may be the source of IL-10 in UMPs living in endemic areas during infection.

## Discussion

In this study, we hypothesized that γδ T cells, which expand in primary or acute infections, do not do so in people with naturally acquired immunity against malaria and focused on the characteristics of these cells in people with naturally acquired immunity against *P. falciparum*. We found that non-Vγ9 γδ T cells, one of the γδ T cell subsets, were elevated in the peripheral blood of UMPs from a malaria-endemic area, although no increase in γδ T cells was observed. In contrast, γδ T cells were significantly higher in patients who were hospitalized with severe malaria than in other groups, and the major subset was Vγ9 γδ T cells. These results should help to resolve the conflicting reports that (i) γδ T cells in patients with primary or acute falciparum malaria expand after antimalarial treatment and this increase persists for 3–4 weeks after treatment (Ho et al., 1990; Roussilhon et al., 1990; Chang et al., 1992; Hviid et al., 1996, 2001; Schwartz et al., 1996; Worku et al., 1997) and (ii) that no increase occurs in γδ T cells in the peripheral blood of UMPs from endemic areas (Goodier et al., 1993; Hviid et al., 1996). We also propose that the two γδT cell subsets, the Vγ9 and non-Vγ9 γδ T cells, play different roles in response to *P. falciparum* infection. Vγ9 γδ T cells, which expand in malaria-naïve individuals but not in partially immune individuals following repeated infection, and these cell populations may be involved in the pathogenesis of malaria. Alternatively, non-Vγ9 γδ T cells, which expand in partially immune individuals, may be involved in conferring protection against malaria and promoting the acquisition of natural immunity after reinfection.

The major γδ T cell subset in the peripheral blood is Vγ9Vδ2, and the other major subset has a Vδ1 chain. From the TCR repertoire analysis, we found that the non-Vγ9 γδ T cells that increased in the UMPs had polyclonal TCR repertoires, with a Vγ2, Vγ3, Vγ4, or Vγ5 chain and a Vδ1 or Vδ3 chain. This finding was consistent with results from a Vδ1 TCR analysis showing that the Vδ1 γδ T cell population, which expands in falciparum malaria patients, uses the whole Vγ chain repertoire and that it is highly polyclonal (Hviid et al., 2001). A polyclonal population of γδ T cells may be necessary to counter the antigenic diversity present in malarial infections (Covell et al., 1953; Healer et al., 2004). Malarial antigens for human γδ T cells have been identified as soluble Pf-schizont-associated antigens, including phosphate antigens (Behr et al., 1996; Pichyangkul et al., 1997) and gametocyte-specific antigens (Ramsey et al., 2002). However, there is little information on non-Vγ9 T cell-specific antigens (Vantourout and Hayday, 2013). We used crude Pf Ag when culturing PBMCs from people living in malaria-endemic area, but the γδT cell response to crude Pf Ag in the presence of IL-2 was slightly different from that in the presence of IL-2 alone. Repeated malarial infections in endemic areas have been reported to be associated with a decreased proportion of Vδ2 γδ T cells in peripheral blood and decreased proliferation and cytokine production in response to malaria antigens (Jagannathan et al., 2014; Farrington et al., 2016). This low responsiveness of γδ T cells to Pf Ag may also be associated with repeated malarial infections in endemic areas. Therefore, more detailed study of the γδ TCR usage and antigen recognition in non-Vγ9 γδ T cells after malaria infection is required.

During malaria infection, γδ T cells bridge innate and adaptive immune responses (Stevenson and Riley, 2004). These cells are stimulated by Pf antigens and proliferate and produce high levels of IFN-γ (Elloso et al., 1994; Pichyangkul et al., 1997; Troye-Blomberg et al., 1999) and other cytokines (Goodier et al., 1995). γδ T cell proliferation and IFN-γ production are enhanced by IL-10, IL-12, and IL-1β (Pichyangkul et al., 1997). A study on the function of γδ T cells during malaria infection showed that these cells are significantly increased in vivax malaria patients from endemic areas with paroxysms and are positively correlated with disease severity (Perera et al., 1994). In contrast, γδ T cells have been reported to play important roles in the elimination of parasites via cytotoxic activity (Roussilhon et al., 1994; Costa et al., 2011), inhibition of RBC invasion by merozoites (van der Heyde et al., 1995), and inhibition of blood-stage *P. falciparum* parasite growth through direct contact (Elloso et al., 1994; van der Heyde et al., 1995; Troye-Blomberg et al., 1999). Similarly to Vγ9 γδ T cells, non-Vγ9 (Vδ1) γδ T cells can also produce IFN-γ when stimulated with Pf Ag (Hviid et al., 2001; D’Ombrain et al., 2007) and inhibit *P. falciparum* growth (Troye-Blomberg et al., 1999). However, the function of non-Vγ9 γδ T cells during malaria infection is largely unknown.

In this study, we revealed that only the non-Vγ9 γδ T cells from people living in malaria-endemic areas proliferated and produced IL-10 in PBMC cultures stimulated with IL-2 and/or Pf Ag for 10 days. We also showed that the plasma levels of IL-10 were elevated only in UMPs from endemic areas and that there was a slight positive correlation between the percentage of non-Vγ9 γδ T cells and the plasma levels of IL-10. These non-Vγ9 γδ T cells also produced IFN-γ. IFN-γ plays a key role in controlling *Plasmodium* infection in both liver and blood stages of the parasite life cycle, but it can also exacerbate the severity of malarial disease depending on its temporal and spatial production (King and Lamb, 2015). The number of IFN-γ-producing γδ T cells among total producing cells is substantially higher in the Vγ9 γδ T cells than in the non-Vγ9 γδ T cells both in JHCs and UMPs. IFN-γ produced by non-Vγ9 γδ T cells in people living in endemic areas may play an important role in the protection of hosts against malaria in the presence of fewer Vγ9 γδ T cells, in agreement with findings from recent reports (Jagannathan et al., 2014; Farrington et al., 2016). IL-10 is an anti-inflammatory cytokine produced by many cell types, including T-helper type 2 (T_H_2) cells, regulatory T cells, T_H_17 cells, CD8^+^ T cells, B cells, dendritic cells, macrophages, mast cells, natural killer cells, eosinophils, and neutrophils (Saraiva and O’Garra, 2010). IL-10 is also an important cytokine for antibody production by B cells and their differentiation into antibody-secreting cells and plasma cells (Arpin et al., 1995; Choi, 1997; Agematsu et al., 1998; Choe and Choi, 1998; Yoon et al., 2009; Weiss et al., 2012). Interactions between γδ T cells and B cells are important for class switching (Wen et al., 1996). γδ T cell clones in mice produce IL-10 (Wen et al., 1998), and human Vγ9 γδ T cells produce IL-10 and support antibody secretion by B cells (Caccamo et al., 2006, 2012). We suggest that the increase in non-Vγ9 γδ T cells and IL-10 production by these cells during malarial reinfection in people from endemic areas plays important roles in the acquisition of natural immunity after an increase in memory B cells and malaria-specific antibodies, although IL-10 is also produced by other cells during malaria infection (Freitas do Rosario and Langhorne, 2012; Stanisic et al., 2014). Together, these results suggest that non-Vγ9 γδ T cells, similarly to other IL-10-producing cells, contribute to the acquisition of natural immunity against *P. falciparum* by stimulating the proliferation and differentiation of B cells into antibody-producing cells after IL-10 production. Surprisingly, non-Vγ9 γδ T cells from NCs living in endemic areas also proliferate in the presence of IL-2 and/or Pf Ag, and they produce moderate levels of IL-10 and high levels of IFN-γ, as compared with the corresponding levels in UMPs *in vitro*. These results indicated that NCs with naturally acquired immunity against malaria-specific antibodies but no detectable parasitemia respond to *P. falciparum* infection and might have more potent naturally acquired immunity than that of UMPs. The differences in cytokine production by non-Vγ9 γδ T cells between UMPs and NCs are thought to depend on the existence of *P. falciparum* parasites in the blood and the immunological status of the patients acquiring natural immunity. Further studies on non-Vγ9 γδ T cells should be performed to evaluate this phenomenon.

There have been few studies on immunity against *P. falciparum* in people living in malaria-endemic areas. To develop an effective malaria vaccine, it is important to determine the factors and mechanisms that lead to the natural acquisition of immunity. Numerous *in vitro* studies have been conducted to elucidate the roles of γδT cells in malaria, especially the Vγ9 γδ T cells, a major γδ T cell subset, but these cells were not found to be increased in UMPs with naturally acquired immunity living in endemic areas. We emphasize the importance of studying non-Vγ9 γδ T cells derived from UMPs with naturally acquired immunity rather than from patients with primary or acute falciparum malaria. Non-Vγ9 γδ T cells, which increase in malaria patients living in endemic areas, may play an important role in the natural acquisition of immunity. Therefore, non-Vγ9 γδ T cells are likely to be important for the development of B cell memory against falciparum malaria. Further studies on these cells should facilitate new approaches for the design of a malaria vaccine.

## Author Contributions

TT performed the research, analyzed the data and wrote the paper; TT, KM, DN, HT, and VV helped with sample and clinical data field collection; CL, MN, and MT provided technical advice; SK supervised field-based research; HW helped perform the research and commented on the draft manuscript.

## Conflict of Interest Statement

The authors declare that the research was conducted in the absence of any commercial or financial relationships that could be construed as a potential conflict of interest.

## Footnotes

1 http://www.who.int/malaria/publications/country-profiles/2008/en/
