# Supplementary material for: A Unique Subset of γδ T Cells Expands and Produces IL-10 in Patients with Naturally Acquired Immunity against Falciparum Malaria
Source: Front Microbiol. 2017 Jul 19;8:1288. doi: 10.3389/fmicb.2017.01288 (PMC5515829; doi:10.3389/fmicb.2017.01288)
Supplement: Supplementary file 1 [file Presentation_1.PDF]

Supplementary Information

A unique subset of γδ T cells expands and produces IL-10 in patients with naturally acquired immunity against falciparum malaria

Tomoyo Taniguchi[1–3*], Kaiissar Md Mannoor[4], Daisuke Nonaka[5], Hiromu Toma[5], Changchun Li[6], Miwako Narita[7], Viengxay Vanisaveth[8], Shigeyuki Kano[9], Masuhiro Takahashi[7], and Hisami Watanabe[3]

*** Correspondence: Tomoyo Taniguchi**: ttani@gunma-u.ac.jp

1 Supplementary Materials and Methods

1.1 Hospitalized clinical samples and healthy controls

Peripheral blood mononuclear cells were isolated from uncomplicated and severe falciparum malaria patients admitted to the Hospital for Tropical diseases, Faculty of Tropical Medicine, Mahidol University, Bangkok, Thailand on the day of admission (day 0), at one week (day 7), and at one-month (day 28). Patients were informed of the rules of the Hospital ethical board, and the research was approved by the Ethics Committee of Faculty of Tropical Medicine. Severe falciparum malaria and uncomplicated falciparum malaria were defined according to WHO guidelines (World Health Organization, 2006). Healthy controls were recruited voluntarily from people who live in non-endemic area of malaria, Bangkok, and had no history of malaria exposure and denied travelling to any malaria-endemic area in the past two years. Their blood smears were proven free of malaria parasites by microscopic examination.

1.2 Flow cytometric analysis

The surface phenotypes of lymphocytes were obtained by three-color immunofluorescence tests. Fluorescein isothiocyanate (FITC)-, phycoerythrin (PE)-, or peridinin chlorophyll protein (PerCP)-conjugated monoclonal antibodies (mAbs) were used. Anti-CD3 (145-2C11), anti-CD4 (SK3), anti-CD8 (SK1), anti-CD5 (L17F12), anti-CD16 (B73.1), anti-CD20 (L27), anti-CD27 (L128), anti-CD38 (HIT2), anti-CD56 (MY31), anti-CD57 (HNK-1), anti-CD138 (MI15), anti-CD161 (DX12), anti-Vα24, anti-IgD (IA6-2), anti-IgG (G18-145), anti-Vγ9TCR (TM-β1), anti-αβTCR (H57-597), anti-γδTCR (GL3) and anti-CD45 (2D1) (BD Biosciences, Mountain View, CA) were used. Cells were examined using a FACSCalibur (BD Biosciences) instrument.

2 Supplementary Figures and Tables

2.1 Supplementary Tables

Table S1: Hematological characteristics of hospitalized uncomplicated and severe falciparum malaria patients and healthy controls.

Characteristic	hUMPs[a] (n=13)	hSMPs[a] (n=11)	HCs (n=12)	*P* Value[b]
Age, median (range)	20.0 (16.0-28)	18.0 (14-26)	25.0 (21-29)	
Sex (Female/ Male)	5/ 8	4/ 7	7/ 5	
WBC/ μL, median (range)	6,200 (3,100-8,500)	8,000 (3,100-12,600)	6,550 (4,500-8,600)	0.1040
% Neutrophil, median (range)	66.0 (33.0-89.0)	73.5 (56.0-95.0)	56.0 (33.0-72.0)	0.0082
% Lymphocyte, median (range)	16.0 (3.0-46.0)	12.5 (0.0-30.0)	30.0 (15.0-50.0)	0.0026
% Monocyte, median (range)	6.0 (0.0-24.0)	11.0 (3.0-18.0)	7.0 (5.0-20.0)	0.3969
% Eosinohil, median (range)	3.0 (0.0-21.0)	1.5 (0.0-3.0)	3.0 (0.0-6.0)	0.0672
% Basophil, median (range)	0.0 (0.0-1.0)	0.0 (0.0-0.0)	0.0 (0.0-1.0)	0.4361
Parasitaemia/ μL, median (range)	8,820 (46.5-145,920)	194,530 (33-823,200)	(-)	0.0277
Microscopic observation (*Pf*)	(+)	(+)	(-)	
α-*Pf* IgG Abs titer, median (range)	595.1 (131.3-7586.9)	312.3 (45.2-3298.9)	0.0	0.0412

Abbreviations: hUMPs, hospitalized uncomplicated malaria patients; hSMPs, hospitalized severe malaria patients; HCs, Healthy Controls; *Pf*, *Plasmodium falciparum*; Abs, antibodies.
[a]Diagnosed using microscopic observation and classified according to WHO guidelines.
[b]Calculated from Kruskal-Wallis test.

Table S2: Plasma levels of cytokines in falciparum malaria patients and negative controls in endemic areas.

Cytokine	UMPs[a] (n=37)	NCs[a] (n=18)	*P* Value[b]
IL-1β, median, pg/mL (range)	44.7 (0.0-137.3)	50.6 (22.9-66.7)	0.2191
IL-2, median, pg/mL (range)	53.9 (0.0-79.9)	58.1 (37.2-70.6)	0.6997
IL-4, median, pg/mL (range)	30.3 (8.0-183.8)	29.0 (15.9-63.9)	1.0000
IL-6, median, pg/mL (range)	41.0 (9.2-190.7)	30.1 (22.7-62.4)	0.8717
IL-8, median, pg/mL (range)	52.0 (19.8-178.9)	54.6 (25.8-501.4)	0.4955
IL-12p70, median, pg/mL (range)	18.0 (0.0-30.1)	19.2 (13.6-24.9)	0.4510
TNF-α, median, pg/mL (range)	22.7 (0.0-59.0)	24.9 (16.1-33.5)	0.0620
IFN-γ, median, pg/mL (range)	24.7 (0.0-61.0)	26.4 (11.2-37.4)	0.5066

Abbreviations: UMP, Uncomplicated malaria patients; NC, Negative Control; NEC, Non Endemic Control; *Pf*, *Plasmodium falciparum*; Abs, antibodies.
[a]Diagnosed using *Pf* rapid test and microscopic observation and excluded Mix (*Pf* and *P. vivax*) infection and Rapid test (+)/ Microscopic observation (-).
[b]Calculated from Mann-Whitney *U* test.

2.2 Supplementary Figures

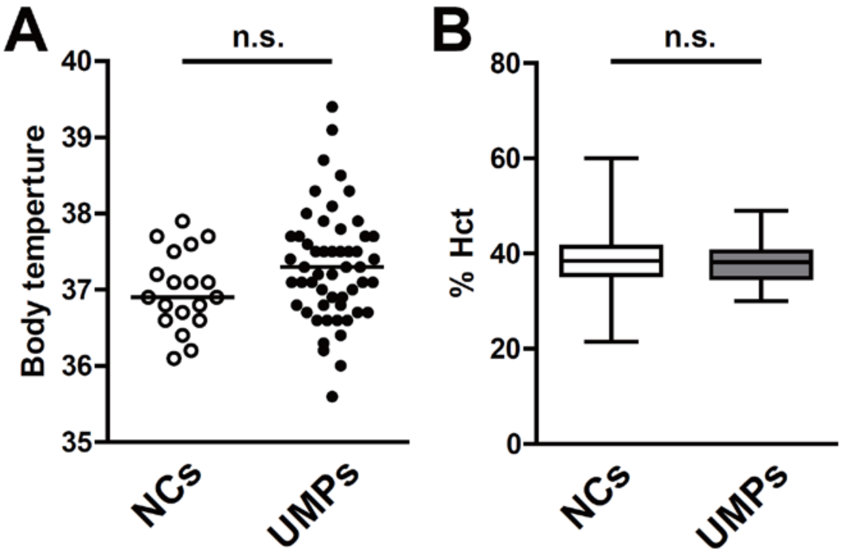

Figure S1: Body temperature and percentage of hematocrit in falciparum malaria patients in endemic areas. Body temperature (°C, A) and percentage of hematocrit (Hct, B) in 19-30 negative controls (NCs) and 26-53 uncomplicated malaria patients (UMPs) in malaria-endemic areas. Statistical analysis was performed using a nonparametric Mann-Whitney *U*-test. n.s.: not significant.

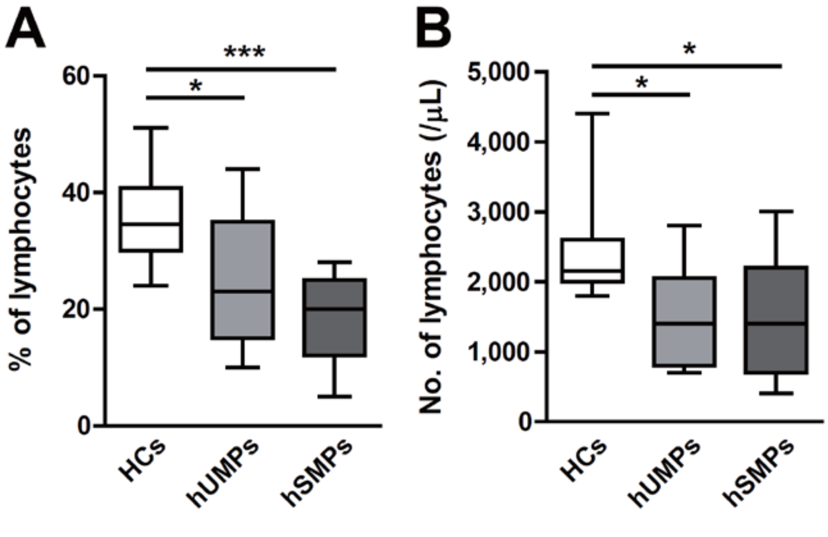

Figure S2: Decrease in the number of lymphocytes following acute falciparum malaria infection. The percentage (A) and absolute number (B) of lymphocytes in 12 healthy controls (HCs), 13 hospitalized UMPs (hUMPs) and 11 hospitalized severe malaria patients (hSMPs). Statistical analysis was performed using a nonparametric Kruskal-Wallis test with Dunn's post hoc test. $^{*}p < 0.05$, $^{**}p < 0.01$, $^{***}p < 0.001$ compared with HCs.

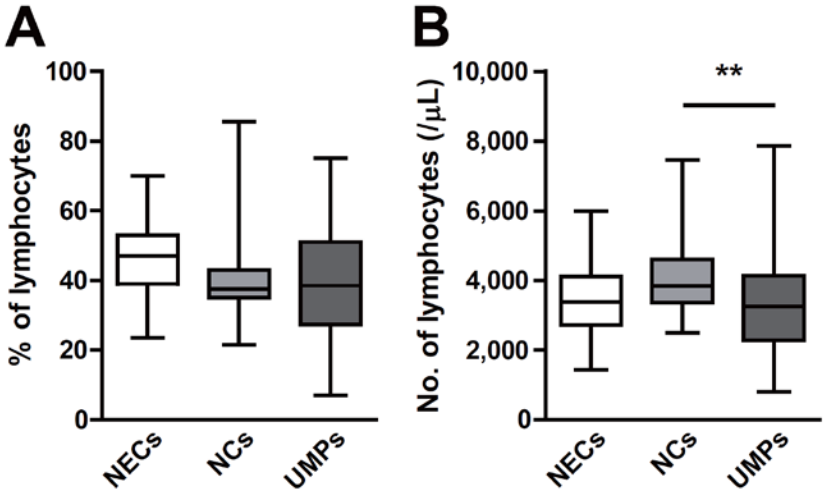

Figure S3: Decrease in the number of lymphocytes from uncomplicated malaria patients living in an endemic area. The percentage (A) and absolute number (B) of lymphocytes in 63 non-endemic controls (NECs), 31 negative controls (NCs), and 91 uncomplicated malaria patients (UMPs) in malaria-endemic areas. Statistical analysis was performed using a nonparametric Kruskal-Wallis test with Dunn's post hoc test. **p < 0.01 compared with NCs.

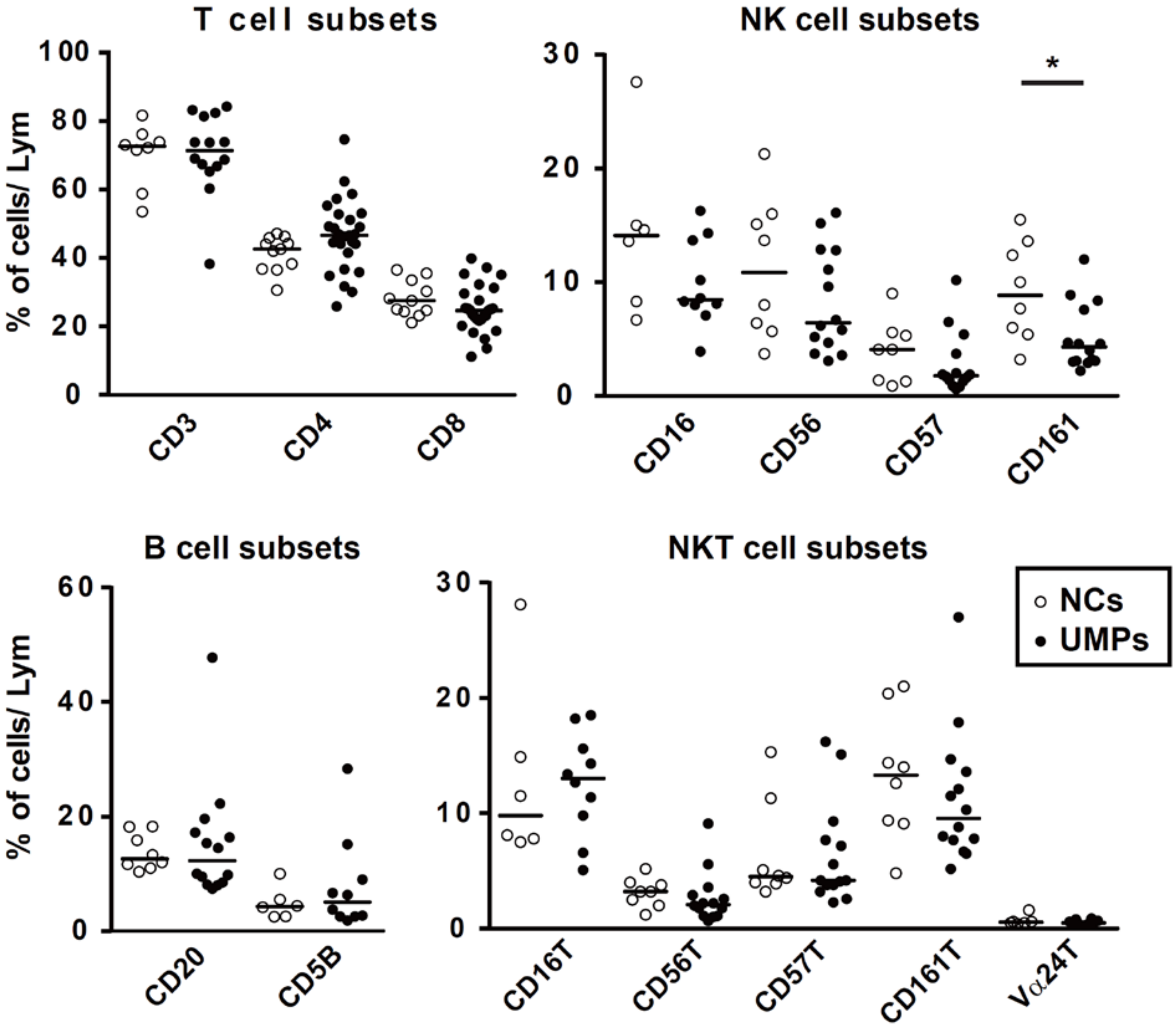

Figure S4: Phenotypic characterization of lymphocytes by flow cytometric analysis in falciparum malaria patients in endemic areas. The proportion of T cell subsets (CD3+, CD4+ T and CD8+ T cells), B cell subsets (CD20+ and CD5+ B cells), NK cell subsets (CD16+, CD56+, CD57+ and CD161+ cells) and NKT cell subsets (CD16+ T, CD56+ T, CD57+ T, CD161+ T and Vα24+ T cells) in 6-11 negative controls (NCs) and 10-20 uncomplicated malaria patients (UMPs) in malaria-endemic areas. Statistical analysis was performed using a nonparametric Mann-Whitney *U*-test. **p* < 0.05.

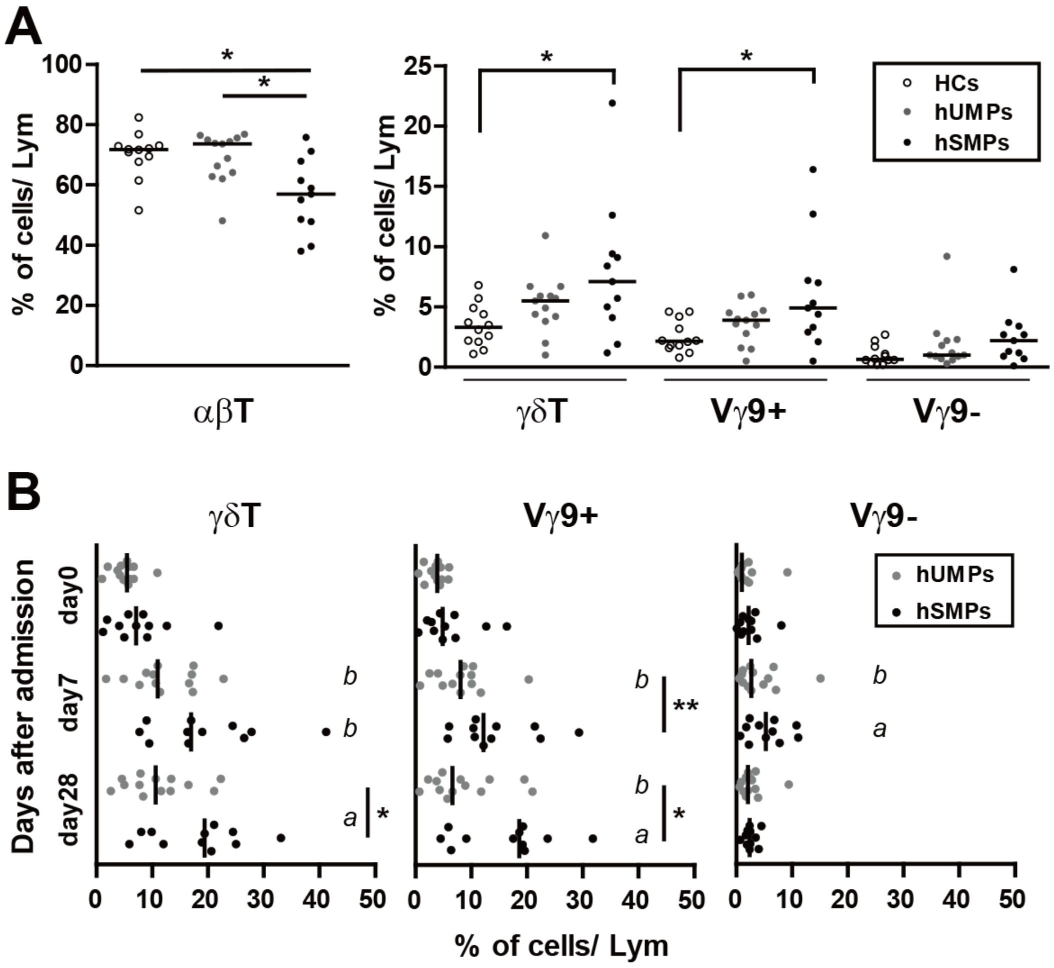

Figure S5: Phenotypic characterization of γδT cells in hospitalized falciparum malaria patients. The proportion of αβT cells and γδT cell subsets on the day of admission (day 0) (A) and the change in the percentage of γδT cells and γδT cell subsets on the day of admission (day 0), at one week (day 7), and at one-month (day 28) in 12 healthy controls (HCs), 13 hospitalized uncomplicated malaria patients (UMPs), and 11 hospitalized severe malaria patients (hSMPs). Statistical analysis was performed using a nonparametric Kruskal-Wallis test and Friedman test with Dunn's post hoc test (A, B) and Mann-Whitney *U*-test (B). *$p < 0.05$, **$p < 0.01$. $^{a}p < 0.05$, $^{b}p < 0.01$ compared with day 0.

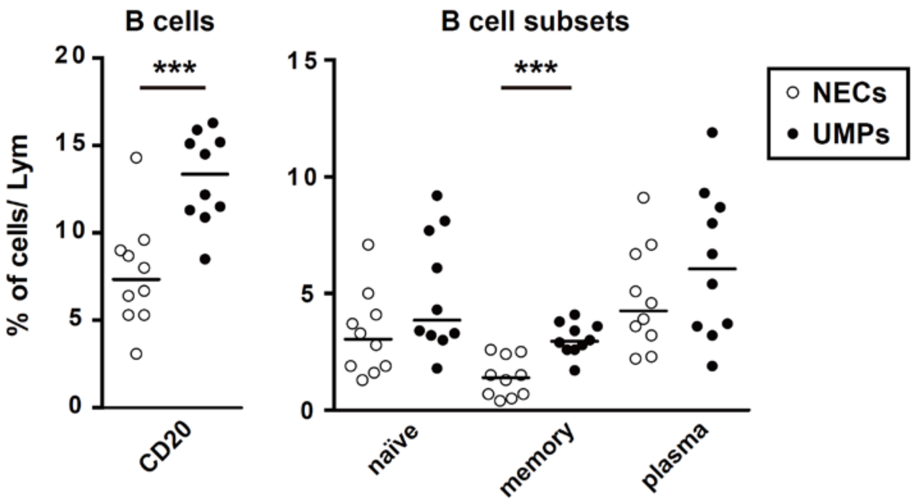

Figure S6: B cell memory in falciparum malaria patients in endemic areas. The proportion of B cell subsets, naïve B cells (IgD+ CD27- CD20+), memory B cells (IgG+ CD27+ CD20+), and plasma cells (CD38+ CD138+) in 10 non-endemic controls (NECs) and 10 uncomplicated malaria patients (UMPs) in malaria-endemic areas. Statistical analysis was performed using a nonparametric Mann-Whitney *U*-test (B). ***p<0.001.

3 Reference

World Health Organization (2006). *Guidelines for the Treatment of Malaria*. Geneva: World Health Organization.
